# Mineral Weathering and Metal Leaching under Meteoric Conditions in F-(Ba-Pb-Zn) Mining Waste of Hammam Zriba (NE Tunisia)

**DOI:** 10.3390/ma16237443

**Published:** 2023-11-30

**Authors:** Oumar Barou Kaba, Fouad Souissi, Daouda Keita, Lev O. Filippov, Mohamed Samuel Moriah Conté, Ndue Kanari

**Affiliations:** 1 Institut Supérieur des Mines et Géologie de Boké (ISMGB), Baralandé, Tamakènè, Boké BP 84, Guinea; 2 Université de Lorraine, CNRS, GeoRessources, F-54000 Nancy, Francendue.kanari@univ-lorraine.fr (N.K.); 3 Institut National de Recherche et d’Analyses Physico-Chimiques (INRAP), Laboratoire des Matériaux Utiles (LMU), Sidi Thabet 2026, Tunisia; souissifoued2@gmail.com; 4 Université de Tunis El Manar, Faculté des Sciences de Tunis, Département de Géologie, Tunis 2092, Tunisia

**Keywords:** Hammam Zriba, F-(Ba-Pb-Zn) deposit, tailings, AMD, metal mobility

## Abstract

Mining waste is an obvious source of environmental pollution due to the presence of heavy metals, which can contaminate soils, water resources, sediments, air, and people living nearby. The F-(Ba-Pb-Zn) deposit of Hammam Zriba located in northeast Tunisia, 8 km southeast of Zaghouan was intensively exploited from 1970 to 1992. More than 250,000 m^3^ of flotation tailings were produced and stored in the open air in three dumps without any measure of environmental protection. Thus, in this paper, mineralogical and chemical characterization, especially the sulfide and carbonate phases, were carried out to evaluate the potential for acid mining drainage (AMD) and metal leaching (ML). Conventional analytical methods (XRD, XRF, SEM) have revealed that this mining waste contains on average 34.8% barite–celestine series, 26.6% calcite, 23% quartz, 6.3% anglesite, 4.8% fluorite, 2.1% pyrite, and 0.4% sphalerite. The content of sulfides is less important. The tailing leaching tests (AFNOR NFX 31-210 standard) did not generate acidic leachate (pH: 8.3). The acidity produced by sulfide oxidation was neutralized by calcite present in abundance. Furthermore, the leaching tests yielded leachates with high concentrations of heavy metals, above the authorized thresholds. This high mobilization rate in potential toxic elements (PTE) represents a contamination risk for the environment.

## 1. Introduction

At the beginning of the mining activity period, the remediation codes for mining companies were less rigorous than those of today [1]. After exploitation, almost all companies leave mine wastes in the direct vicinity of the mine [2]. In the 1800s and 1900s, most mines had no laws governing the management and control of mining waste, which was a source of pollution for decades [3]. Many of these wastes contain harmful components in concentrations that can present serious hazards to the ecosystem and human health [4]. According to Eyankewere and Obasi [5], 62% of heavy metals contained in water and soils come from mining, 23% from indiscriminate waste disposal, and 15% are attributed to diverse anthropogenic and geogenic activities.

About 50 deposits were exploited in Tunisia, mostly from the end of the nineteenth century, and there are about 600 mineral occurrences identified in the northern part of the country. The primary deposits, which are veins, impregnations, and replacement orebodies, are located within upper Cretaceous to Tertiary sedimentary rocks. The element association consists of Pb-Zn-Fe-Ba-F, with Cd, As, Sb, and other elements serving as subordinates. Carbonates (calcite and dolomite) make up the majority of the gangue minerals, with silica, clays, and gypsum appearing less frequently [6,7,8,9,10,11]. Many abandoned tailings resulting from the exploitation of these deposits within the district of Zaghouan (NE Tunisia) have been the subject of environmental impact studies [11,12,13,14,15,16,17]. The studies of these former mining wastes were conducted according to different disciplines, such as mineralogy, geochemistry, heavy metal mobility, and environment (soil, atmosphere, and watercourse contamination).

In a semi-arid Mediterranean context, the extent of contamination by mining wastes may be worsened by: (i) violent climatic events, such as high-speed winds and annual rainfall concentrated into just a few months [18,19,20,21]; (ii) the lack of vegetation cover, which favors the mobilization of tailing particles loaded with metals [21].

The most costly and serious environmental problem associated with mining and milling metallic ores is considered to be acid mine drainage (AMD) and associated metal leaching (ML), which can last for hundreds or even thousands of years [22,23]. Once an acid environment has been established, other sulfide, oxide, silicate, and carbonate minerals dissolve and release, and depending on the nature of the ore deposit, metals such as Cu, Zn, Pb, Ni, Cd, Co, Hg, Al, Mn, and U and metalloids including As, Sb, and Se [24]. These elements present a mobility that constitutes a potential risk of pollution for soils and sediments as indicated by Doufexi et al. [25], Alexakis et al. [26], and Bu et al. [27].

The exploitation of the F-(Ba-Pb-Zn) deposit of the Zaghouan district (north-eastern Tunisia) has generated more than one million tons of mine (flotation) tailings (Hammam Zriba, Hammam Jedidi, Jebel Ressas) [11,12,13,14,15,16,17], stored in the open air and exposed to atmospheric oxygen and rainfall. According to Lettermoser [28], metals, metalloids, acid, and salts released into tailing pore waters as a product of oxidation and weathering reactions can: (a) be retained within the tailings impoundment; (b) enter surface and groundwater systems; or (c) be precipitated at the particulate level of the tailings impoundment. According to earlier research, Cu may precipitate as covellite beneath the zone of sulfide oxidation to create a zone of copper enrichment [29,30]; or massive precipitation of secondary minerals including oxides, sulfates, and sulfides occurs at specific levels within the tailings impoundment [31,32]. In this paper, the authors determine grain size distribution, chemical, and mineralogical composition, and carry out leaching tests as a function of rainfall conditions in a semi-arid context, to assess the environmental impact of the studied mine tailings.

### 1.1. Location and Weather Conditions of the Study Area

**Figure 1:**
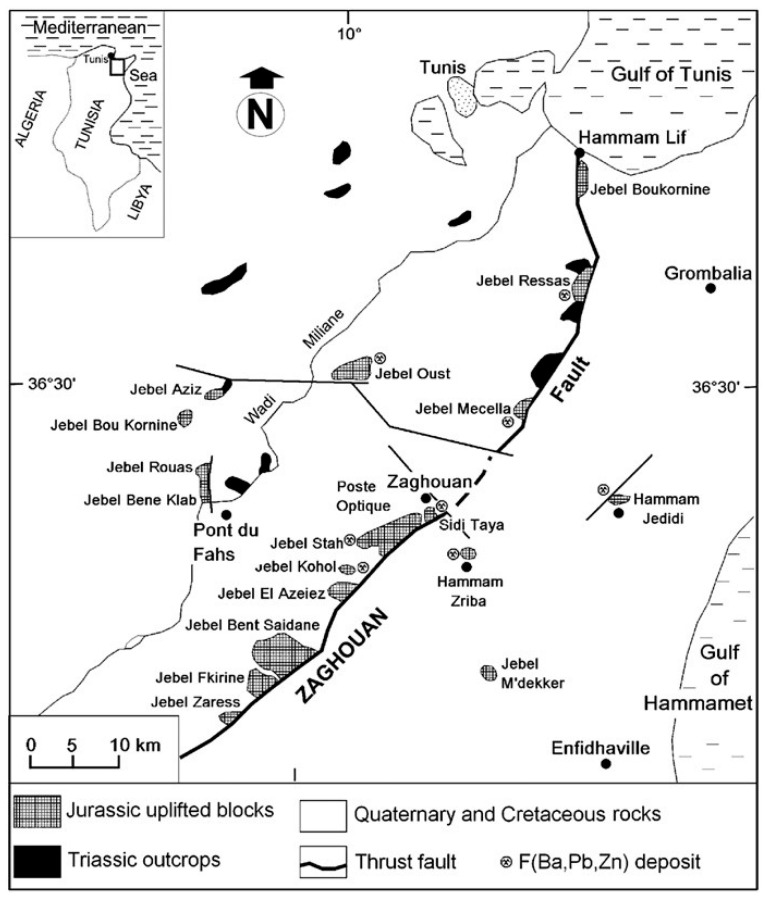
Location map showing the Jurassic uplifted blocks and Triassic outcrops, along with F-(Ba-Pb-Zn) deposits, in north-eastern Tunisia [33].

The Hammam Zriba mine is located in north-eastern Tunisia, 60 km south of Tunis, and 8 km southeast of Zaghouan (Figure 1). The reliefs of the region culminate at 360 m.

**Figure 2:**
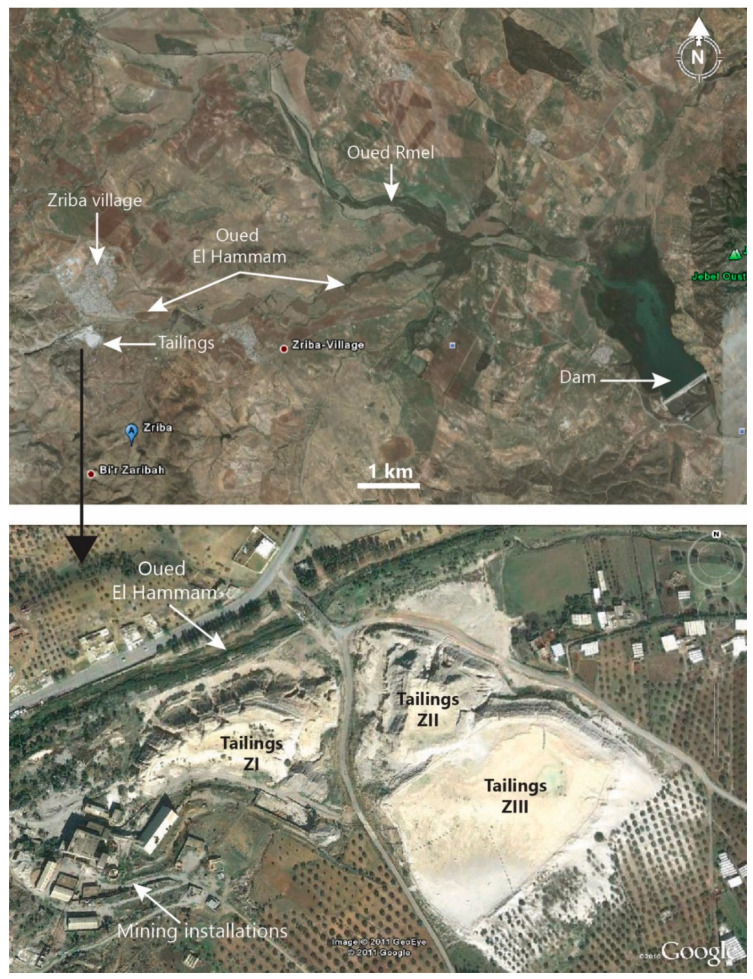
Hammam Zriba tailings location https://www.google.com/maps/@36.3423656,10.208982,492m/data=!3m1!1e3?entry=ttu/ (accessed on 28 July 2011).

Figure 2 shows the three tailings dikes located on the banks of the Oued Hammam, which flows into the Oued R’mel dam located 12 km downstream from the mining site.

Hammam Zriba as well as all this region has a semi-arid Mediterranean climate. At Hammam Zriba (1998–2008), the average annual rainfall ranges from 180 to 820 mm. Higher and lower rainfall are recorded during January and August, respectively. The highest temperatures (33 °C) are observed in July and August, while the lowest temperatures (6 °C) are recorded during December, January, and February. The prevailing wind direction is northwestern with an average annual speed of 3.5 m s^−1^ [34].

### 1.2. Geological Setting

**Figure 3:**
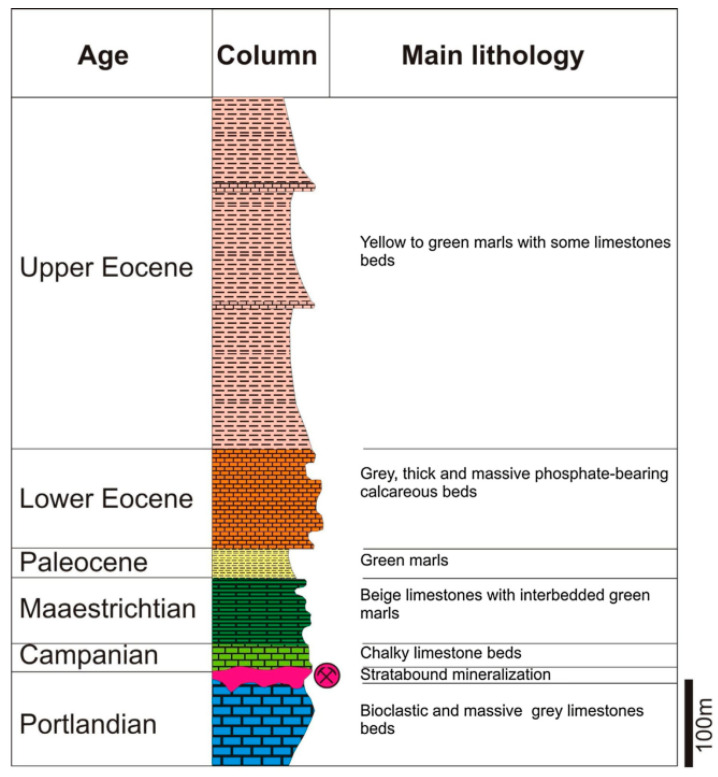
The lithostratigraphic column of the Hammam Zriba deposit modified from Melki and Zargouni in Jemmali et al. [39] (Adapted and reprinted with permission from Melki and Zargouni, 1991).

Like many deposits identified in the NE of Tunisia, the Hammam Zriba deposit is located along the great Zaghouan fault (Figure 2). The lithostratigraphic column at Hammam Zriba from the bottom to the top consists of a stratigraphic sequence including the Tithonian (Upper Jurassic) and Oligo-Miocene (Tertiary) series (Figure 3), in which the Tithonian is unconformably overlain by the Middle-Upper Campanian [9,35,36].

Tectonics in horsts, grabens, and tilted blocks provoked important palaeogeographic variations, responsible for lateral changes in facies, condensation of sedimentation or emersion, and unconformities [37,38].

The mineralization in the Hammam Zriba deposit is formed by a 0 to 8 m thick stratum level, following the unconformity that separates the Late Jurassic (Tithonian) limestones, making the so-called “Ressas Formation” and the Late Campanian limestones with fine marly intercalations. According to Bouhlel et al. [9], the ore consists mainly of barite–celestite series (40–45%), fluorite (15–35%), quartz (10–40%), and galena and sphalerite (5–15%) in carbonated gangue. The formers may be accompanied by small amounts of pyrite, chalcopyrite, bornite, phosphate, and greenockite. Other oxidation and accessory minerals can be present, such as anglesite, cerusite, smithsonite, chalcocite, gypsum, malachite, azurite, hemimorphite, iron hydroxides, native sulfur, and clay minerals.

## 2. Materials and Methods

### 2.1. Sample Collection

**Figure 4:**
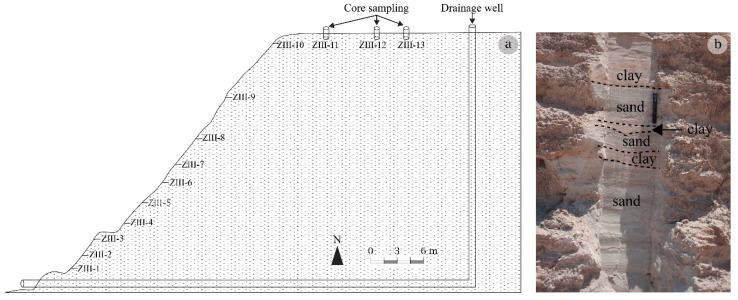
Flotation tailings of Hammam Zriba: (**a**) sampling positions, (**b**) surface variation in texture and color.

The beneficiation of Hammam Zriba ore generated more than 280,000 m^3^ of tailings [40] divided into three piles, labeled from the oldest to the youngest ZI, ZII, ZIII, and dumped over 96 m^2^ [41]. Thirty-seven samples were collected from these three dumps: 12 samples from ZI, 10 samples from ZII, and 15 samples from ZIII. Samples were taken after clearing the fresh surface on the dumps’ sides (Figure 4) and also through PVC tube cores and on the dumps’ top surfaces. Each sample collected weighed about 1 kg and was put in a polyethylene bag. The dumps consist of gray to light gray materials, consisting of centimeter- to decimeter-thick layers extending horizontally of the flotation tailings, having a fine- to coarser-grained texture and diverse colors (gray, green, yellowish), interbedded with thin clay layers (Figure 4). Perforations, apparently due to living organisms observed in large numbers only in the ZI dump, reveal a possible form of life in this mine waste. The loose character of the material constituting the dumps facilitates their erosion and particle transport by runoff (hydric) towards Oued Hammam, giving rise to pullying on the dump flanks.

**Figure 5:**
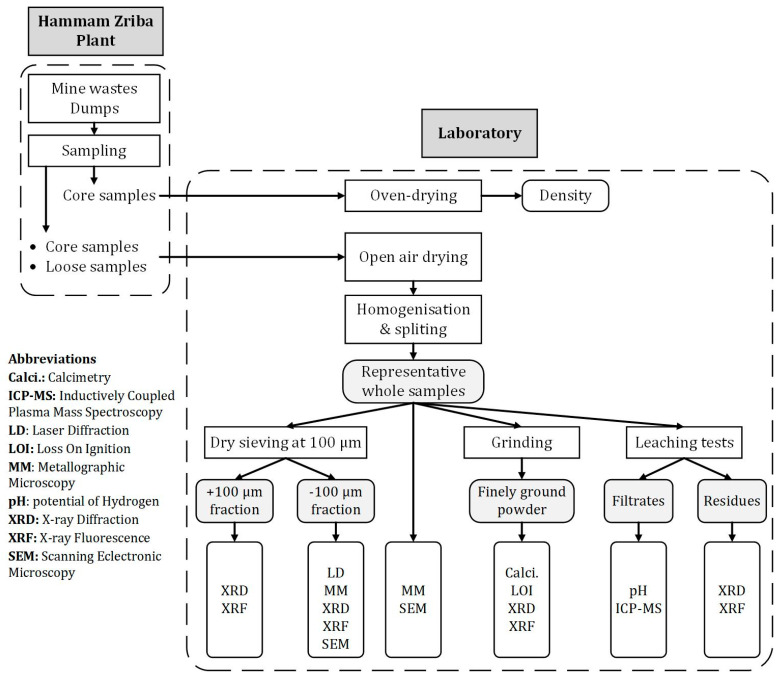
Outline of the methodology used in this study.

Samples were dried in open air and then homogenized and quartered to obtain representative samples for different analyses (Figure 5). Wet sieving at 100 µm was performed on the representative sample, and the −100 µm fraction was exclusively analyzed using microscopy methods because of their relatively high sulfide content. For analyses requiring powder samples (XRD, XRF, wet chemistry), a part of the grinding was carried out in an agate mortar and the other part was done using a laboratory disc mills manufactured by SIEBTECHNIK TEMA company (Rijswijk, The Netherlands) in tungsten carbide bowls.

### 2.2. Apparent Density

The apparent density determination of mine wastes was performed on core samples taken by using 22.8 cm long metal cylindrical tubes with an inside diameter of 5 cm. After sampling, the cores were weighed before and after drying in an oven for 48 h at 60 °C. Calculations were made using the following formulas:

(1) $$\rho =\frac{M}{V}\;and\;V=\pi r^{2}h$$

where M is the core mass, V is the core volume, r is the core radius, and h is the core length.

### 2.3. Grain Size Analysis

The particle size distribution was determined using laser diffraction with Sympatec HELOS with short bench-type BF and equipped with a helium–neon laser light source (632.8 nm) at Laboratoire Environnement et Minéralurgie (LEM become LIEC, Nancy, France). Analysis was carried out without and then with ultrasound for a 120 s duration.

### 2.4. Mineralogical Analysis

The qualitative mineralogical analysis of the major phases in the mine waste was determined by X-ray diffraction on a Bruker D8 instrument using CoKα radiation of wavelength 1.78026 Å. The X-ray beam was operated at a voltage of 35 kV and a current intensity of 45 mA. The diffraction patterns were processed using DIFFRAC. EVA V4 software supplied by Bruker AXS Gmbh (Karlsruhe, Germany) for peak identification with a PDF database. TOPAS software V5 supplied by Bruker AXS Gmbh (Karlsruhe, Germany), which takes into account the Rietveld method, was used for quantitative mineralogical analysis [42,43]. The XRD analysis was carried out at the Laboratoire Environnement et Minéralurgie (LEM, Nancy, France). For calcium carbonate content, the Bernard calcimeter volumetric method was employed at Laboratoire des Ressources Minérales et Environnement (LRME, Tunis, Tunisia).

The particle morphology and texture were investigated using microscopy analysis, specifically an Olympus metallographic microscope BX51M (Tokyo, Japan) and a HITACHI 500 Scanning Electron Microscope (SEM) (Shibuya City, Japan) equipped with a Si-Li X-ray detector and a backscattered electron detector (KE Development). Energy-Dispersive Spectroscopy (EDS) allows the local semi-quantitative analysis of a large range of elements. These analyses were conducted on polished thin sections (prepared by embedding the loose mine waste sample in resin) and on non-polished thin sections (prepared by sticking particles on the sample holder). These investigations were performed at Laboratoire GeoRessources, Nancy, France.

### 2.5. Chemical Analysis

The chemical composition of the whole sample was determined using mobile Energy Dispersive X-ray Fluorescence spectroscopy (ED-XRF) with a Niton XLt (USA) at Laboratoire GeoRessources, Nancy, France. Sample preparation consisted of making a pellet of 3 cm diameter of finely ground powder sample without a binder in a polyethylene mold. Chemical analyses were also performed using a laboratory ED-XRF at Laboratoire d’Electrochimie des Matériaux (UMR-CNRS 7198, Metz, France). Trace elements in the solutions were analyzed using Inductively Coupled Plasma Mass Spectroscopy (ICP-MS) (Perkin Elmer Elan 6000, PerkinElmer Inc., Waltham, MA, USA) with multi-elementary calibration and two quality controls (International standard river water (SLRS-5) and Volvic water). An Ion 450 MeterLab millivoltmeter equipped with a specific electrode was used to analyze fluorine. The ICP-MS analysis was performed at the Service des Roches et des Matériaux (SARM-CNRS, Nancy, France).

The loss on ignition (LOI) for the determination of the weight percent of organic matter and carbonate content in mine waste was carried out by oven-drying 1 g of the sample at 105 °C for 24 h to remove retained water, and organic matter was transformed into ash and carbon oxide between 500 and 550 °C during the second step of sample heating at 1000 °C for 3 h in a muffle furnace. After cooling in a desiccator and weighing the calcined, the LOI was calculated using the following formula:

(2) $$LOI=\frac{DW_{105}-DW_{1000}}{DW_{105}}\;*\;100$$

with $DW_{105}$: dry weight at 105; $DW_{1000}$: dry weight at 1000.

### 2.6. Leaching Test

To investigate the behavior of minerals contained in the mining waste under surface conditions, leaching tests were carried out on a representative whole sample of each one of the three dumps. Leaching tests were performed according to standard AFNOR NF X31-210 [44]. Each sample was mixed with deionized water (with 18 ΩM resistivity) using a solid-to-water ratio of 1:10. A mass of 100 g was suspended in 1 L of deionized water in a glass bottle. The mixture was shaken using a horizontal shaker HS 501 D manufactured by IKA company (Staufen, Germany) at 60 rpm for 24 h to allow a uniform sample suspension. A double filtration was performed on each suspension. First, a slow filtration was carried out through Wahtman filter paper number 42, then the filtrates were re-filtrated on a 0.45 µm cellulose membrane. The total duration of these two filtration steps was below 30 min. All the above operations were performed at room temperature.

For metal analysis, the filtrates were acidified and trace elements were analyzed using ICP-MS. The filter residues were dried in an oven at 103 °C for 24 h, weighed, and analyzed for chemical and mineralogical compositions.

The pH of suspensions and filtrates was measured with a pH 3110 WTW pH meter (calibrated with standard solutions of pH 4.01, 7.00, and 10.00).

## 3. Results

### 3.1. Physical Properties of Mining Waste

#### 3.1.1. Particle Size Distribution

The grain size distribution is the fundamental property of tailings that controls the permeability [45]. Particle size analysis performed using the dry sieving method shows that the three tailings dumps are constituted essentially of sand with a proportion of fine fraction (−100 µm) greater than 40% (Table 1).

Dump ZI (the oldest) is much finer with 63.3% fine sand compared to dumps ZII and ZIII, which contain 46.7 and 40.1%, respectively. The coarse sand proportion in the three dumps is less important: 2.3, 2.1, and 4.0% for ZI, ZII, and ZIII, respectively. The predominance of fine sand resulted from grinding operations carried out to reach the release mesh of the minerals during the initial valorization (fluorite and barite).

**Figure 6:**
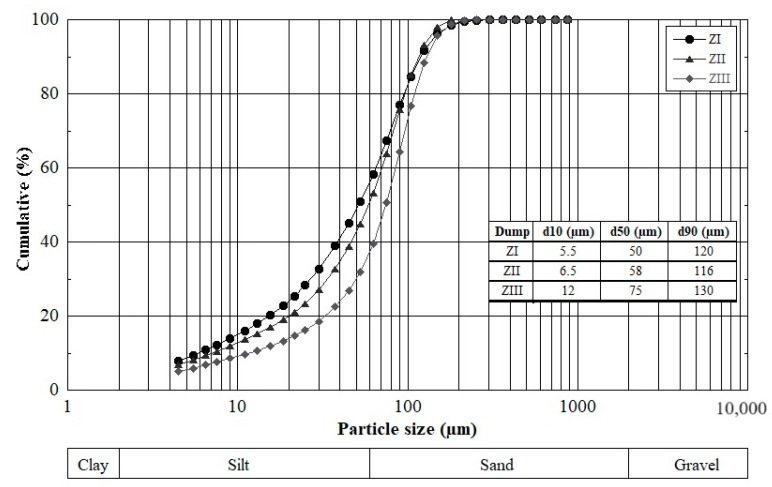
Particle size distribution of HZMW (−100 µm fraction) using laser light scattering.

Laser light scattering analysis of the −100 µm fraction shows a particle size distribution dominated essentially by silt (2–63 µm) and fine sand (63–100 µm), with d_90_ ranging from 116 to 130 µm (Figure 6).

The fine particle size can reach 4.5 µm, which easily predisposes these particles to wind transport and also gives them a high reactivity related to their specific surface area being relatively larger than that of coarse particles. According to their −100 µm fraction content, the three dumps can be ordered as follows: ZI > ZII > ZIII. The three waste dumps present a monomodal or homogeneous grain size distribution with a mean size (d_50_) centered around 50–75 µm.

#### 3.1.2. Apparent Density

Table 2 shows the densities of the main minerals present as well as those of the wet and dry apparent densities of the studied wastes, which are equal to 3.18 and 3.11 g/cm^3^, respectively. Such densities show that the flotation tailings contain high amounts of gangue (low density) minerals (calcite and quartz).

### 3.2. Mineralogical Characterization

**Figure 7:**
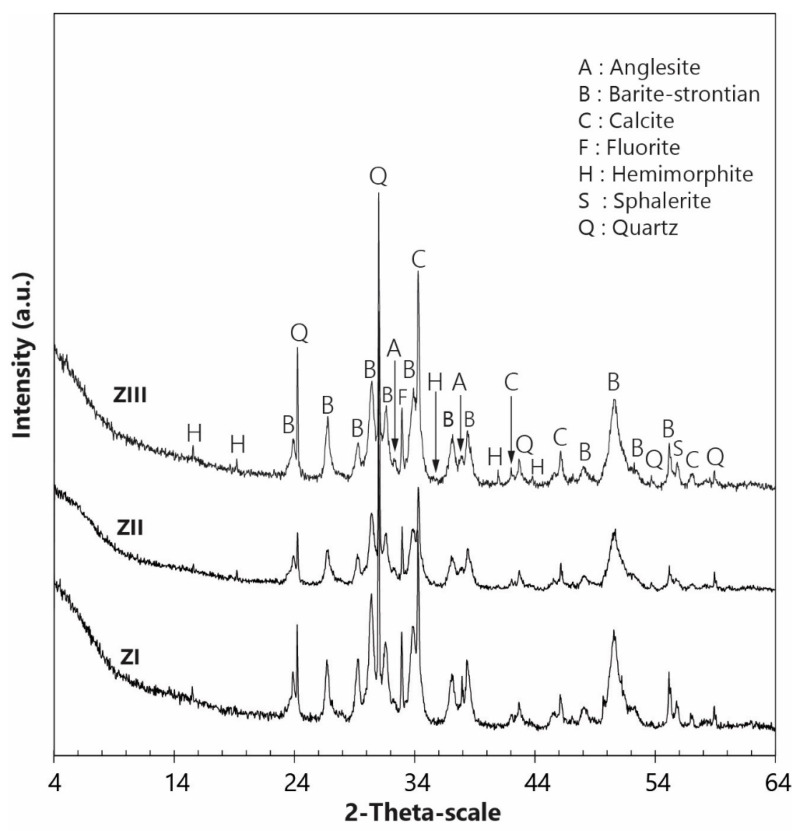
XRD patterns of Hammam Zriba tailings.

The mineralogy of Hammam Zriba mining waste, determined by XRD analysis, is dominated by barium-rich (strontian-barite) minerals associated with calcite, quartz, fluorite, anglesite, hemimorphite, sphalerite, and pyrite (Figure 7).

**Figure 8:**
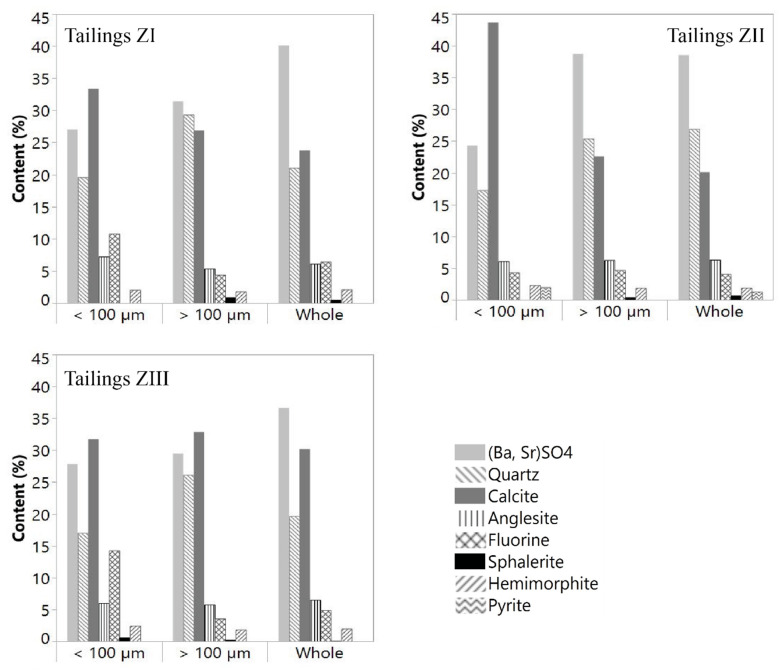
Quantified mineralogical composition of Hammam Zriba tailings.

Strontian-barites and barian-celestites constitute a series of solid solution ranging from barite (BaSO_4_) to celestite (SrSO_4_). Bouhlel showed [46] that barian-celestite and strontian-barite are more abundant than pure barite or pure celestite. The strontian-barite mineral series contents determined using TOPAS software V5 are 40.1, 38.6, and 36.6% in the tailings ZI, ZII, and ZIII, respectively. (Ba, Sr)SO_4_ contents in the −100 µm fraction range from 24.3 to 27.8% while those in the +100 µm fraction are between 29.5 and 38.7% (Figure 8).

The three tailings’ contents of quartz and calcite, the main gangue minerals, range from 19.5 to 26.9% and from 20.1 to 30.2%, respectively. The percentages of fluorite in whole samples from dumps ZI, ZII, and ZIII are 6.4, 4.1, and 4.9%, respectively. Fluorite content (main ore mineral) reaches 14% in the −100 µm fraction of the dump ZIII, which is made of the more recent tailings. Moreover, analysis reveals the presence of notable amounts of anglesite (6.1–6.5%) and hemimorphite (1.9–2.1%). The sulfides identified by XRD were sphalerite (0.1–0.8%) with pyrite (1.3%) but only in the dump ZII. This value increases slightly to 2% in the −100 µm fraction.

**Figure 9:**
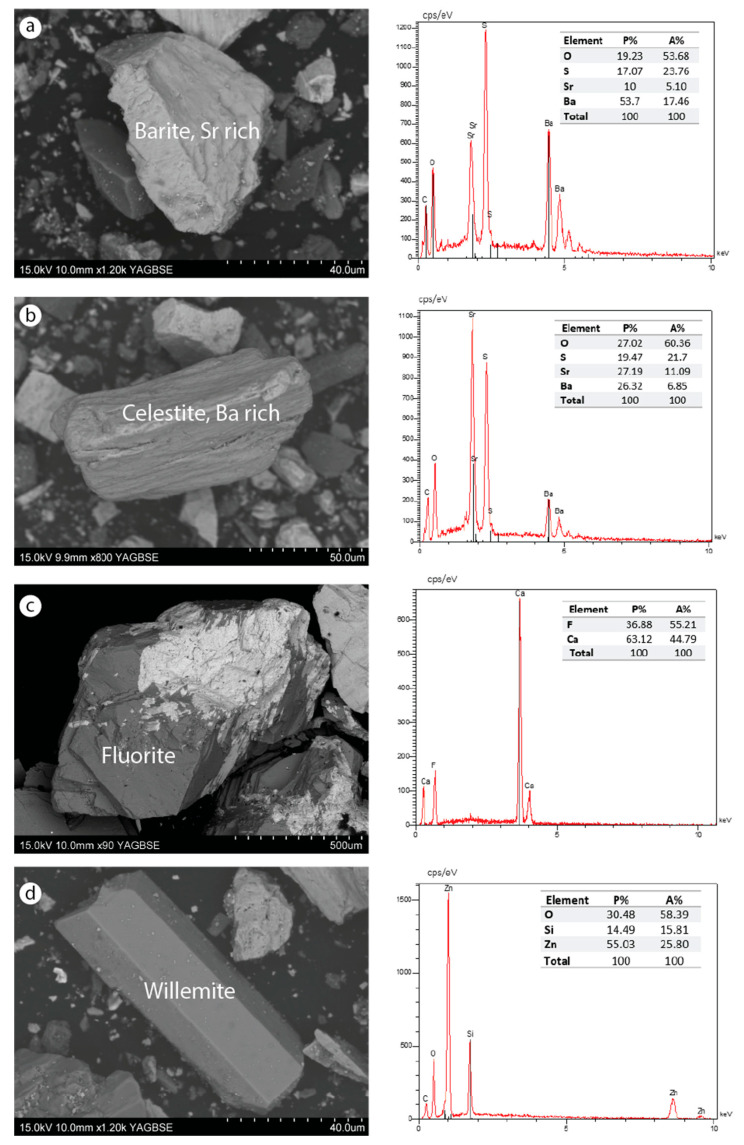
SEM images of a sample from the dump ZIII. Released grain: (**a**) strontian-barite (Bar, Sr-rich); (**b**) barian-celestite (Cel, Ba-rich); (**d**) willemite. Mixed grain: (**c**) fluorite and strontianite.

**Figure 10:**
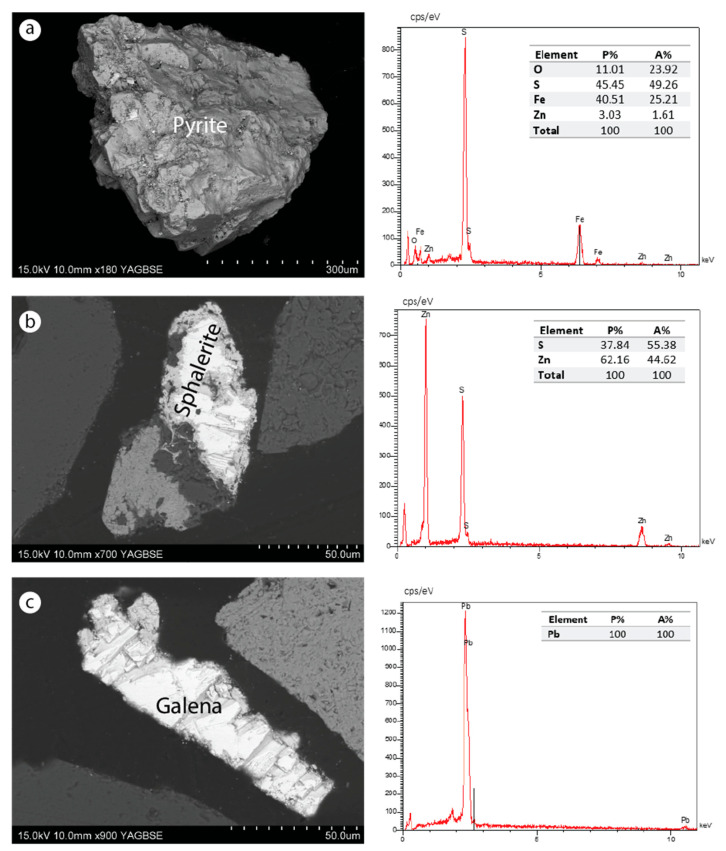
SEM images of sulfide minerals identified in the dump ZIII. (**a**) Fully released pyrite grain; (**b**) sphalerite combined with quartz and barite minerals; (**c**) completely released lead unaltered.

**Figure 11:**
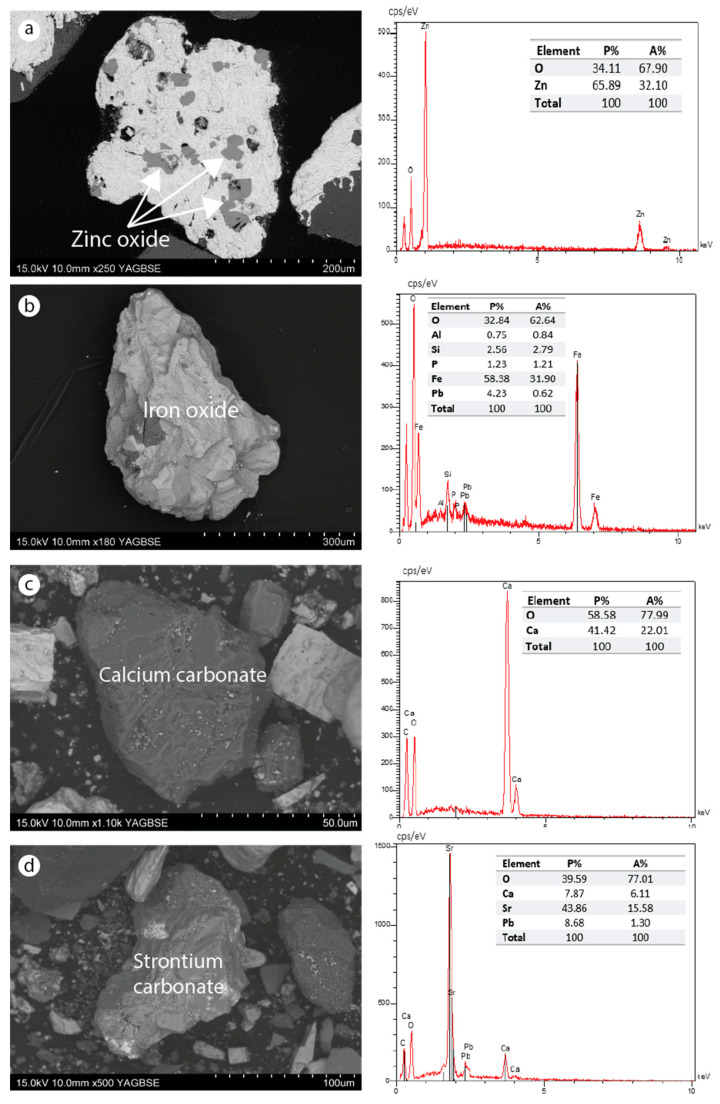
SEM images of oxides and carbonate minerals identified in the dump ZIII. (**a**) Zinc oxide combined with barite; (**b**) iron oxide released containing certain elements in small proportions (Al, P, Pb, and Si); (**c**) carbonate calcium released; (**d**) strontium carbonate with lead.

SEM–EDS analysis was carried out only on the tailings from dump ZIII. The analysis showed irregularly shaped particles, sometimes with well-defined faces (Figure 9, Figure 10 and Figure 11). This irregular morphology of the grains probably results from the mechanical treatments undergone by the ores during the beneficiation process. The main mineral phases are in the form of carbonate, sulfate, silicate, fluorite, sulfide, and oxide. These minerals are found either in released or associated (combined) form. The strontian-barites and barian-celestites, in fibrous and lamellar textures, respectively, are the most abundant minerals. Fluorite was also identified but in a lower proportion. Carbonates and silicates belonging to the gangue are sometimes Zn, Sr, Ba, or P-bearing minerals, such as willemite, cerussite, fluorapatite, or strontium carbonate. Galena, pyrite, and sphalerite are sulfide minerals found in low quantities. Zinc oxide filling the barian-celestite cavities was also identified. Iron oxide probably derived from pyrite alteration, which is indicative of oxidizing environments, was observed in both free and combined forms.

Figure 10 shows the sulfide minerals identified in the dump ZIII. These sulfides are present either in free or combined grains with other minerals, such as quartz or barite.

The main carbonate and oxide minerals identified are shown in Figure 11. Zinc and iron oxides may result from the weathering of sulfides present in the tailings. The carbonates present may contribute to the neutralization of ADM and the stabilization of metals by forming more stable compounds.

### 3.3. Granulochemical Characterization

The chemical analysis was carried out using ED-XRF on the whole sample, as well as on the −100 and +100 µm fractions (Table 3).

SO_3_ is the dominant oxide with contents between 22.92 and 25.29%. The predominance of SO_3_ is related to the high proportion of the (Ba, Sr)SO_4_ mineral series in the tailings investigated. The BaO contents vary from 11.85 to 12.95% and the SrO contents between 5.7 and 6.17%. Calcium oxide (CaO) (15.52–20.44%) and silica (SiO_2_) (18.94–19.57%) constitute the main oxides of the silico-carbonated gangue. Pb and Zn expressed as PbO (<1%) and ZnO (1.64 to 2.23%) are the most abundant heavy metals. The low Fe_2_O_3_ content (<1%) suggests a very low potential for acid mine drainage. Other major oxides were present with low values, such as Al_2_O_3_ (1.40–1.50%) and P_2_O_5_ (2.53–2.62%).

The ED-XRF analysis shows an important procession of heavy metals (Table 4), where Pb (<0.38%) and Zn (1.18%) are the most abundant trace metal elements (MTEs). The −100 µm fraction contains the highest concentration of Pb, Ba, Sr, and As. The fineness of these metal carriers is due to ore-grinding during mechanical preparations. MTE accumulation in the mines can become troublesome if their higher specific surface accelerates the reactions, producing unstable compounds. The fineness of these elements can also facilitate their particulate transport, thus amplifying their pollutant impact.

The contents of these MTEs exceed their limit value according to CCME [47], which makes the studied tailings a potential contamination source.

### 3.4. Leaching Tests

The leaching tests performed on the mine waste of the three tailings dumps, ZI, ZII, and ZIII, showed a mass loss of leached raw samples (13.4, 1.63, and 2.5%, respectively) and a slight decrease in the initial solution pH from 9.45–8.8 to 8.17–8.3 at the end of the test (Table 5). The mass loss reflects the solubilization of certain mineral phases. The pH decreases but remains in the alkaline range, providing evidence that the dissolution of gangue minerals has certainly contributed to neutralizing the acidity generated by sulfide oxidation. Several works [49,50] found similar results and explained that the buffering of acidity is caused by the reacting gangue minerals (carbonates. silicates, and hydroxides).

The soluble fractions (f_o_) from the three dumps each equal 0.001 g kg^−1^, indicating that the soluble amounts are low. This could be a significant factor in the immobilization of heavy metals contained in the mining waste. Table 6 shows the chemical composition of leachate (only for dump ZIII) and filtering residues. Sr and Zn show high dissolution rates in the ZIII leachate of 10,047.14 and 751.26 µg L^−1^, respectively. The ZIII leachate contains 159.29 µg L^−1^ in Ba, which is widely above the limit value authorized for water (700 µg L^−1^). Fe, As, and Hg concentrations in the leachate are lower than the limit of detection; they are therefore present in negligible quantities. Blowes and co-workers [51] indicated that, in mine tailings, acid production occurring as the result of sulfide oxidation generates leachates with high concentrations of dissolved constituents (SO_4_, Fe^2+^, other metals), which may gradually seep from tailings dumps into adjoining surface-water and groundwater flow systems.

Leaching test results (Table 6) show a Ba and Cr content in residue after leaching higher than those present in raw tailings before leaching. This behavior was also identified in [52], specifically for As and Cd, where a soil-contaminated leaching test was carried out according to AFNOR NF X31-210.

The fluoride ion concentration determined using a specific electrode is 3300 µg L^−1^. This value is widely above the WHO requirement of 1500 µg L^−1^ (Table 6). This high fluoride content may result from the alkaline pH of the leachate since fluorite is highly soluble under such conditions. Wenzel and Blum [53] and Zhang et al. [54] found that fluorite is slightly soluble at pH 6.0–6.5, but its solubility increases at both pH < 6.0 and pH > 6.5. According to Wenzel and Blum [53], the high solubility of fluorite under acidic conditions can be explained by the occurrence of cationic complexes, while the increase in fluorite solubility at pH > 6.5 results from the desorption of free fluoride due to repulsion by more negatively charged surfaces.

By referring to limit values, it can be deduced that the contents of all the elements measured in the leachates exceed their respective toxicity limits. The solubilization of these elements shows that the bearing minerals are not completely stable under the physicochemical conditions of the tests, that is, such minerals present in the studied tailings behave as potential sources of water pollution under surface conditions. In their study of the chemical and mineralogical characterization of the weathering products from an abandoned Pb/Zn mine, Khelfaoui et al. [55] report the presence of significant concentrations of certain heavy metals (Pb, Zn, Fe, Mn, Cd, and Hg) in the soil due to the leaching of waste rock and tailings. Perlatti et al. [56] indicate that high concentrations of metals contained in tailings remain unchanged even during long periods of exposure; however, the action of weathering and erosion causes a gradual release of these metals into the environment, thus constituting a source of pollution for the latter.

The X-ray diffraction method did not identify a new crystallized phase in the residues from the leaching tests. However, X-ray diffractograms show a decrease in the intensity of calcite reflections after the leaching tests.

**Figure 12:**
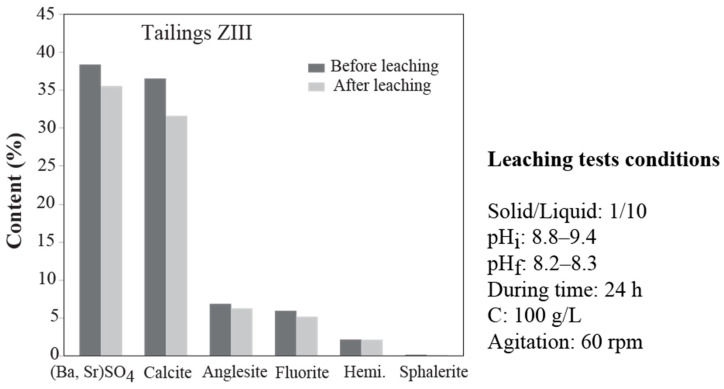
Mineralogical composition of Hammam Zriba ZIII tailings before and after leaching test.

The comparison of mineralogical compositions obtained using TOPAS software V5 shows a decrease in mineral content after the leaching tests (Figure 12).

Calcite and barite–celestite mineral series present the highest decrease rate after the leaching test. Meanwhile, the lowest decrease rates are recorded in the minerals anglesite, fluorite, hemimorphite, and sphalerite. This low proportion decrease may indicate a slight mobilization rate of metallic elements under these conditions, such as Pb, Zn, Ba, and Sr. This low mobilization may be due to the alkaline pH of the medium (8.8–9.4), which is not very destabilizing for the minerals carrying these metallic elements. Souissi et al. [15] indicated that in northern Tunisia mine tailings, Ca^2+^, Mg^2+,^ and SO_4_^2-^ are dominant ionic species, while Pb, Zn, and Cd have lesser dissolution rates.

The heavy metal mobility observed at alkaline pH may be explained by the presence of mineral-bearing of these elements in soluble form under these pH conditions. Indeed, Cappuyns et al. [57] also found a significant leaching rate for heavy metals (Pb, Cu) at pH 10 and argued that this would be due to the atmospheric conditions, which favor the formation of hydroxyl complexes at alkaline pH. The formation of soluble anionic species may also lead to a significant heavy metal release under alkaline conditions [57]. Several studies cited by Krol et al. [58] on waste material show that the heavy metal (cation) leaching curve is U- or V-shaped, with a minimal pH value at around 9. The solubility of Zn, Pb, and Cd contained in tailings is highly influenced by Pb–Zn minerals (sphalerite, smithsonite, galena, anglesite) [57].

Several approaches can be considered to mitigate or significantly reduce the harmful impacts of heavy metals. These approaches include stabilization of the heavy metals in soils, phytoremediation to recover heavy metals from the soil, use of plants to block heavy metals from spreading, or mitigation by using chemical immobilization materials like agricultural limestone, mineral rock phosphate, or diammonium phosphate as experimented by Basta and McGowem [59].

## 4. Conclusions

The tailings generated by the F-(Ba-Pb-Zn) ore processing at Hammam Zriba and stored in dumps, contain high concentrations of metals potentially toxic, such as Ba, Sr, Pb, Zn, Cd, As, and Sb. This study focused on the behavior of metal(loid)-bearing minerals contained in mining waste under meteoric conditions. Mineralogical analysis reveals that these elements are in the form of sulfates (barite–celestite, anglesite), sulfides (pyrite, galena, sphalerite), oxides (iron oxide, zinc oxide), and silicates (hemimorphite, willemite). The PTE-bearing minerals are hosted within a gangue composed mainly of calcite and quartz. Carbonate and silicate wastes are considered non-acid-generating. Their dissolution leads to an increase in pH, which in turn minimizes/prevents the AMD.

Despite the alkaline pH conditions of the test, the leachates contain heavy metal concentrations significantly above the recommended limits, which reveals the mobilization potential of these PTEs under surface conditions.

Although there is no AMD generated by the studied tailings, the mobility of significant quantities of heavy metals represents a real risk of contamination for the surrounding farmlands and water resources, and thus for the health of people living within the vicinity.

## Figures and Tables

**Table 1:** Particle size distribution of Hammam Zriba mining waste (HZMW) by sieving.

Fraction (µm)	Proportion by Dump (%)		
	ZI	ZII	ZIII
500–2000 (Coarse sand)	2.3	2.1	4.0
100–500 (Medium sand)	34.4	51.2	55.9
0–100 (Silt, fine sand)	63.3	46.7	40.1
Total	100	100	100

**Table 2:** Density of Hammam Zriba mining wastes (HZMW) and main minerals contained.

Mineral	Quartz SiO_2_	Calcite CaCO_3_	Fluorite CaF_2_	Hemi. (*)	Sphalerite Zns	Celestine SrSO_4_	Barite BaSO_4_	Pyrite FeS_2_	Anglesite PbSO_4_	Galena PbS	HZMW
Density	2.65	2.71	3.18	3.47	3.9	3.97	4.5	5.02	6.38	7.58	3.11/3.18

(*): Hemimorphite Zn_4_Si_2_O_7_(OH)_2_H_2_O.

**Table 3:** Chemical composition (wt%) of Hammam Zriba flotation tailings.

Sample		CaO	SiO_2_	Al_2_O_3_	Fe_2_O_3_	P_2_O_5_	MnO	MgO	PbO	SO_3_	BaO	SrO	ZnO	LOI
LOD (mg/kg)		50	-	500	35	250	60	3500	5	70	35	3	8	-
ZI	Whole	15.52	18.94	1.50	0.48	2.59	0.26	<LOD	0.26	25.29	12.85	6.17	1.64	9.18
	+100 µm	18.36	20.65	1.32	0.44	2.32	0.23	<LOD	0.19	22.80	11.32	5.44	1.73	11.35
	−100 µm	12.95	17.39	1.63	0.51	2.90	0.27	<LOD	0.33	27.88	14.97	6.93	1.56	7.33
ZII	Whole	16.60	19.53	1.84	0.67	3.20	0.28	<LOD	0.26	27.69	13.56	6.28	1.71	9.54
	+100 µm	17.95	19.21	1.40	0.43	2.62	0.27	<LOD	0.41	24.55	12.80	6.16	2.13	9.00
	−100 µm	15.31	23.15	1.48	0.76	2.78	0.26	<LOD	0.33	25.37	12.19	5.98	2.20	10.13
ZIII	Whole	11.25	21.08	1.77	0.49	3.57	0.27	1.31	0.56	30.17	15.26	7.43	2.22	7.04
	+100 µm	13.91	24.03	1.83	0.52	3.41	0.28	<LOD	0.41	28.38	13.67	6.62	2.27	8.91
	−100 µm	20.44	19.57	1.45	0.41	2.53	0.26	<LOD	0.34	22.92	11.85	5.70	2.23	11.32

**Table 4:** Pb, Zn, Ba, Sr, and trace metal element concentrations (mg kg^−1^) in the flotation tailings of Hammam Zriba mining wastes.

Elements	LOD (mg/kg)	ZI			ZII			ZIII			Limit Value
		Whole	+100 µm	−100 µm	Whole	+100 µm	−100 µm	Whole	+100 µm	−100 µm	CCME [47] WHO [48]
As	3	217.29	117.03	305.04	227.44	197.58	337.18	192.11	261.37	357.72	6
Ba	35	115,075.3	101,382.1	134,096.7	114,634.5	109,150.0	136,637.9	106,153	118,075.7	131,246.0	210
Cd	8	25.34	29.40	25.78	27.14	37.75	33.58	<LOD	28.78	<LOD	0.6
Co	20	255.77	156.29	<LOD	147.25	<LOD	184.45	144.44	204.05	178.56	50
Cr	20	603.13	586.56	667.87	588.52	548.74	618.42	451.04	521.31	551.60	26
Cu	12	128.64	141.57	142.45	149.65	<LOD	181.43	119.76	<LOD	165.08	16
Mo	3	14.58	13.88	12.34	14.32	14.98	18.27	16.26	20.33	19.20	-
Nb	3	17.30	14.28	14.24	16.90	18.14	17.67	16.99	20.39	21.70	-
Ni	25	347.76	311.54	321.90	381.59	427.17	382.73	417.97	436.54	400.24	40
Pb	5	2435.64	1735.77	3104.24	3825.16	3061.27	5159.71	3126.34	3756.08	4687.71	31
Sr	3	52,175.54	45,986.01	58,594.55	52,127.67	50,589.49	62,791.94	48,204.28	53,176.03	59,482.48	240
V	10	<LOD	<LOD	<LOD	618.69	789.73	928.54	901.04	1065.39	1127.89	-
Zn	8	13,138.86	13,884.64	12,569.27	17,142.92	17,714.76	17,828.89	17,891.32	17,458.82	18,974.48	120

**Table 5:** Parameters and results of leaching tests conducted on the Hammam Zriba mine waste.

Dump	m_o_ (g)	m_s_ (g)	pH_i_	pH_f_	v_o_ (l)	v (l)	C (g L^−1^)	f_o_ (g kg^−1^)
ZI	100	97.27	9.45	8.17	2.7·10^−3^	1	100	0.001
ZII	100	98.49	9.35	8.25	1.5·10^−3^	1	100	0.001
ZIII	50	48.75	8.8	8.3	1.2·10^−3^	0.5	100	0.001

m_o_: mass before leaching; m_s_: dry residue mass at 105 °C; pH_i_: pH before leaching; pH_f_: pH after leaching; v_o_ = 10^−3^(m_o_ − m_s_): volume of initial water contained in the sample; v: volume of water used for the test; C: concentration of leachate; f_o_: soluble fraction of raw wastes = C(v + v_o_)/m_o_.

**Table 6:** Chemical composition of leachate and residues of Hammam Zriba mining waste (Dump ZIII).

Elements	Whole Sample (mg kg^−1^)		Leachate (µg L^−1^)		Residues * (mg kg^−1^)	Limit Value [47,48]	
	Value	LOD	Value	LOD		Water (µg L^−1^)	Sediment (mg kg^−1^)
Al	7674.06	500	96.28	1.77		200	
As	192.11	3	-	0.59	81.44	10	6
Ba	106,153	35	159.29	0.06	112,670.67	700	210
Cd	<LOD	8	8.85	0.003	<LOD	3	0.6
Cr	451.04	20	12.74	0.11	3541.84	50	120
Cu	119.76	12	22.39	0.21	75.52	2000	16
F	-	-	3300	-	-	1500	
Fe	2871.50	35	-	8.26	941.86	200	
Mn	1975.54	60	15.59	0.09	1355.69	100	-
Ni	417.97	25	4.4	0.16	<LOD	70	16
Pb	3126.34	5	23.91	0.02	-	10	31
Sr	48,204.28	3	10,047.14	0.03	29,261.94	1500	240
Zn	17,891.32	8	751.26	0.26	8351.73	9030.83	8351.73

LOD: Limit of Detection; * LODs are identical to those indicated for the whole sample.

## Data Availability

The data presented in this paper are available on request from the corresponding author.
